# Barriers to sexual and reproductive health communication in Southwest Ethiopia: perspectives of parents, youths, and teachers

**DOI:** 10.3389/frph.2025.1444603

**Published:** 2025-05-13

**Authors:** Abraham Tamirat Gizaw, Sualiha Abdulkader Muktar, Yohannes Addisu Wondimagegene, Mengistu Abayneh, Belay Zeleke Sefere, Kasahun Girma Tareke, Abebayehu N. Yilma

**Affiliations:** ^1^ Department of Health, Behavior and Society, Faculty of Public Health, Institute of Health, Jimma University, Jimma, Ethiopia; ^2^ Health System Department, International Institute for Primary Health Care, Addis Ababa, Ethiopia; ^3^ College of Health Sciences and Medicine, Dilla University, Dilla, Ethiopia; ^4^ School of Medical Laboratory Science, Jimma University, Jimma, Ethiopia; ^5^ Early Childhood Care and Education Specialist, Bonga Education College, Bonga, Ethiopia; ^6^ Department of Public Health Sciences, College of Medicine, Pennsylvania State University, Hershey, PA, United States; ^7^ Institute of Energy and the Environment, Pennsylvania State University, University Park, PA, United States

**Keywords:** barriers, sexual, reproductive health, parents, youths, teachers

## Abstract

**Background:**

Youths throughout the world face considerable challenges related to their sexual and reproductive health (SRH). In Ethiopia, the adolescent and youth groups account for nearly half the population. Parents play a vital role in SRH communication. Parents’ communication with their children regarding SRH is considered an important part of adolescent development, as this contributes to optimizing safe SRH. Therefore, this study aimed to explore barriers to SRH communication from the perspectives of parents, youths, and teachers in Southwest Ethiopia.

**Methods:**

A qualitative research approach was used in four schools (two private and two public) in Jimma town. Data collection entailed 16 focused group discussions with parents and youths (15–24 years) and 12 key informant interviews with school directors, unit leaders, and school media coordinators. The interviews were audio-recorded, transcribed, and translated into English. Thematic analysis was conducted using ATLAS_ti software.

**Results:**

Participants recognized the crucial role parents play in SRH issues; however, only a few parents were involved in SRH communications, and there were many barriers raised by the participants. Barriers to SRH communications were parental lack of knowledge, cultural and religious beliefs, the effect of social media use, shame and stigma, and parental attitudes towards SRH communication.

**Conclusions:**

In summary, addressing barriers to sexual and reproductive health communication between parents and youth is crucial for fostering better health outcomes. Parents should be encouraged to have open discussions with their children from an early age. High schools should implement regular SRH education sessions with healthcare providers, while youth-friendly service clubs should focus on changing attitudes towards SRH communications through social and behavioral changes in communication.

## Background

Sexual and reproductive health (SRH) is the integration of emotional, physical, intellectual, and social wellbeing concerning sexuality and it is not merely the absence of dysfunction (1, 2). SRH communication refers to interpersonal communication about SRH issues that takes place between parents, friends, and/or significant others (3). The health threats for youth today are more behavioral than biological and more of today's youth behaviors involve serious consequences (4, 5).

Although most youth want to know about abstinence, contraception, and how to prevent HIV/AIDS and other sexually transmitted infections, parents often have difficulty communicating about SRH issues (6, 7). Youth-parent SRH communication is affected by social norms and taboos related to gender and sexuality (8). These factors create a culture of silence, particularly for young girls, in asking, obtaining information, discussing, and expressing their worries about SRH (9). The United Nations’ Sustainable Development Goals incorporated the issue in Goal Three of promoting a healthy lifestyle. Following this, at the country level, Ethiopia launched a national reproductive health strategy plan (10) and the Adolescent and Youth Health Strategy. Furthermore, Ethiopia's public health facilities, including health centers and health posts, provide fee-exempted SRH services, despite the shortage of medical supplies (11).

Parent-youth SRH communication is very poor, particularly in developing countries. Some of the reasons found were that parents perceived their children as too young to be discussing this topic and that it is culturally forbidden (12). In addition, parents wrongly perceive that communicating SRH issues with youths promotes early sexual intercourse. Furthermore, youths also did not prefer to access necessary information from their parents, but they were eager to gain information from peers/friends and social media (13). To improve youth SRH status in Ethiopia, the government has implemented several frameworks during the past 23 years. Moreover, young people have the highest rate of unmet need for contraception; they are at high risk for HIV infections, with 40% of newly diagnosed HIV infections occurring in the 15–24-year-old age group (14).

Despite policies, initiatives, and strategic measures aimed at increasing youth utilization of SRH services in Ethiopia, their utilization remains very low (15). According to the 2019 Ethiopian Demographic and Health Survey, 62% of youths initiated sexual activity before the age of 18, and by age 20, 36% of men had engaged in sexual practices (13). Such early sexual activity exposes them to SRH-related problems due to receiving inappropriate information from various media or friends. Excessive media exposure without parental support on these sensitive issues, particularly SRH, has negatively affected youths. Consequently, parents have a responsibility to engage in open discussions about SRH issues with their children, as youths are highly vulnerable to health problems, particularly in the area of SRH (14).

A study conducted in Nekemtie showed that 43.8% of youths prefer to receive SRH information from schoolteachers. The finding of this study suggests that empowering schoolteachers with appropriate SRH information and strengthening SRH clubs in schools can boost youth awareness and understanding of SRH issues (16). The National Adolescent and Youth Health Strategy (2021–2025) clearly stated that training and sensitizing teachers and families on age-appropriate information, behavioral change communication tools, and parenting roles is a priority area to address SRH issues in Ethiopia (11). Even though there are many strategies and policies implemented in Ethiopia, detailed studies are still required to explore barriers that hinder SRH utilization and communication among different stakeholders. Therefore, this study aimed to explore barriers to sexual and reproductive health communication in Jimma town, Southwest Ethiopia.

## Methods

### Study design and setting

A descriptive qualitative study design was conducted in Jimma town, located 357 km from Addis Ababa and with an estimated population of 207,573. The town is home to 16 high schools, 7 of which are public and 9 are private. Altogether, these schools educate 19,074 youths, comprising 9,123 boys and 9,951 girls. Additionally, youth-friendly service clubs and organizations such as family guidance associations are present in the town, offering support to youths by providing reproductive health information and services.

### Study population

The study population was parents of youths, teachers, school directors, unit leaders, and school media coordinators. Youths were recruited from different grades and schools. Parents were recruited using the registered school contact information, and teachers were also recruited from different schools. The key informant interviews (KIIs) were conducted with the school directors, unit leaders, and school media coordinators. The participants and data sources were chosen in stages to saturate ideas about parent-youth sexual and reproductive health communication. The study applied a purposive sampling technique for data collection (Table 1).

**Table 1:** Summary of the conducted FGDs.

Participants	Jimma University Community School	Jimma Preparatory School	Catholic High School	Eldan School
Parents				
Fathers only	1	1	1	1
Mothers only	1	1	1	1
Youths				
Boys only	1	1	1	1
Girls Only	1	1	1	1

### Sampling

A multistage stratified sampling technique was employed to select two private and two public schools. Students from grades 9–12 were chosen using computer-generated random numbers in Microsoft Office Excel. Parental contacts were gathered from the schools, and then parents were selected based on gender using computer-generated random numbers in Microsoft Excel 2016. Additionally, school directors, unit leaders, and media coordinators were purposively selected.

### Data collection method

Focus group discussions (FGDs) and KIIs were the methods used for qualitative data collection. FGDs were used to capture a wide range of views and enable interaction between participants with differing experiences regarding parent-adolescent communication, which provided greater insight into attitudes, perceptions, beliefs, and practices. We conducted 16 FGDs (eight with parents and eight with youths). The FGDs were disaggregated by sex to allow for free expression of views during the discussion of potentially sensitive issues (Table 1). A total of 12 KIIs were conducted with school directors, unit leaders, and media coordinators, three from each school.

### Data collection tools

Semi-structured interview guides (FGDs guide for parents and youths, and KIIs for school directors, unit leaders, and media coordinators) were developed. The interview guides were based on a review of the literature, field experience, and research objectives. The areas explored included youths’ and parents’ knowledge, attitudes, practices; barriers to parent-youth communication; experiences and perceptions of SRH communication; and preferred sources of SRH. All interviews (which took 40 and 92 min, respectively) were documented with a voice recorder, and minutes were taken at every interview. The interviews were carried out in Afan Oromo and Amharic.

### Data validation

Lincoln and Guba's criteria were used to ensure the validity of the data (17). Initially, the data collection tool (interview guide) was pre-tested. A research assistant and a team engaged in peer debriefing to establish trustworthiness. After consulting with the lead investigators, the tool was modified. To diversify the study participants, youths attending school and key informants were recruited based on sociodemographic characteristics, such as gender, age, and education level, to gather a wider range of perspectives. A summary of significant themes was presented to the study participants at the end of each data collection day, and a discussion was held to clarify any confusing findings. The transcription and translation were provided to the participants, along with a summary of key themes and challenging concepts, so that they could review the interpretations and provide their comments, critiques, explanations, and confirmation.

Prolonged engagement, achieved by spending an extended period in the research area, was undertaken. The lead investigators verified the points mentioned in the interviews throughout this time. To ensure transferability, the entire research process, participants’ different viewpoints and experiences, methods, interpretation of results, and contributions of research assistants were thoroughly described. The dependability of the findings was ensured by the contributions of the participants to the findings, interpretations, and recommendations. The findings of this study have been reviewed and confirmed with colleagues familiar with qualitative research.

### Data processing and analysis

The data from the interviews were transcribed verbatim and then translated into English for analysis. ATLAS.ti 7.1.4 aided in the coding and further analysis of the data. Investigators read and reread the transcripts before assigning codes (open coding) and developing an initial coding structure. Two coders were involved to minimize any bias in the coding process. One coder performed iterative rounds of open coding on selected transcripts to ensure that the coding structure was relevant and appropriate. In contrast, the second coder reviewed and verified the emergent codes.

Finally, the study team analyzed the coded transcripts and generated codes to agree on the coding system and code definitions used to code all transcripts. The findings were categorized into themes and sub-themes based on important quotes. For reporting qualitative findings, this study adheres to the Consolidated Criteria for Reporting Qualitative Research standard protocol (18).

### Ethical consideration

An ethical clearance letter was obtained from the ethical review board of Jimma University’s Institute of Health. The letter was submitted to the Jimma Town Education Department for permission. The respondents were informed, and written consent was obtained. For youths younger than 18 years, consent from the parent or legal guardians was secured. The respondent's right to refuse or withdraw from participation at any time was fully respected, and the information provided by each respondent was kept confidential by coding each questionnaire and not sharing the personal information of any participants with a third party.

## Results

### Study participants’ background characteristics

As shown in Table 2, most of the parents were in the age range of 45–54. However, all the youths were in the age range of 15–24 according to the WHO age classification of youths. Most of the parents were Orthodox Tewahedo Christianity followers, completed primary schooling, and were married. Regarding the teachers, all had completed tertiary education.

**Table 2:** Background characteristics of the in-depth interview, key informant interview, and focus group discussion participants.

Category	Parents	Youths	Teachers
Sex			
Male	26	32	6
Female	28	30	6
Age			
15–24	0	62	0
25–34	8	0	4
25–44	17	0	3
45–54	26	0	4
55 and above	3	0	1
Religion			
Orthodox Tewahedo	18	22	3
Muslim	16	19	7
Protestant	13	11	2
Catholic	7	10	0
Educational status			
None	2	0	0
Primary	19	0	0
Secondary	18	0	0
Tertiary (diploma, degree, and above)	15		12
Marital status			
Single	7		2
Married	42		10
Divorced	5		

### Barriers to sexual and reproductive health communication

Based on the participants’ views, barriers to SRH communication are grouped into the main themes, namely, lack of knowledge, cultural and religious beliefs, excessive social media use, shame and stigma, and parental attitudes towards SRH (Table 3).

**Table 3:** Themes and sub-themes.

Themes	Sub-themes
Lack of knowledge	Limited education on sexual health Lack of comprehensive sex education Misconceptions about reproductive health Lack of access to resources
Cultural and religious beliefs	Discomfort discussing sensitive topics Religious teachings
Effect of social media use	Social media information Need for privacy Media portrayal of sex Excessive social media use
Shame and stigma	Fear of embarrassment Fear of judgment Lack of confidence
Parental attitudes towards SRH	Judgmental attitudes Parental apprehension Lack of openness to dialogue

### Parents’ lack of knowledge

Many parents found it challenging to discuss SRH with their children due to a lack of knowledge, skills, and confidence. They expressed the need for more accessible and age-appropriate information to help them start the discussion. This lack of confidence and knowledge hinders their ability to initiate discussions, as they fear not having the correct information. Some parents had the knowledge but still lacked the confidence to discuss SRH issues with their children. Children also noted that their parents seemed uninformed about SRH, which discouraged them from taking the issue to their parents. Two FGD participants said the following:

“I don’t know much more about sexual and reproductive health (SRH) issues than my young children do because today's youth have access to many sources of information. I’ve never discussed these topics with my children, and to be honest, I’m afraid to bring them up.” (Parent, Male, 61 years, FGD)

“I often advise my son to study hard to achieve good results in his education, but sexual and reproductive health issues are not his concern at this time.” (Parent, Female, 44 years, FGD)

### Cultural and religious beliefs

SRH issues are assumed to be veiled by culture and religion, making it difficult for many people to discuss them openly in the community. Communication concerning SRH issues is considered taboo, making youths feel humiliated when addressing them with their parents. As a result, youths do not receive adequate information about their SRH needs and problems from their parents. In this study, cultural and religious beliefs were the most commonly reported barriers to parent-youth SRH communications. Furthermore, many parents expressed similar thoughts to the youths, therefore, parents avoid the discussions due to the belief that it is taboo. They view SRH issues as private and assume that youths will learn about them without any direct discussion with their parents. One of the FGD participants said

“Sexual and reproductive health issues are often restricted by cultural and religious beliefs. Many parents feel uncomfortable discussing SRH topics and may be unwilling to engage in open communication about these matters with their children.” (Youth, Male,17 years, FGD)

### Effect of social media use

Excessive exposure to social media is negatively affecting communication between parents and youths regarding SRH. Globalization has made it challenging for parents to protect their children from social media influences, particularly Facebook and TikTok. Participants in the study noted that these platforms have made it difficult for parents to engage in meaningful discussions with their children about SRH topics, as they are often overshadowed by misleading information and influences from social media sources. Youths tend to trust and emulate what they see and hear on social media or other media outlets, such as television, more than what their parents tell them. This is how the participants described it. One of the KII participants said

“…It seems that not many parents have discussed SRH issues with their children; instead, they prefer to access information from smartphones and can easily find it online.” (School director, Female, 37 years, KII)

Another KII participant stated that

“…Ideally, youths are too young for us, but they are being highly exposed to an outside culture which contradicts our culture. It creates an individualized environment between parents and youth…” (School media coordinator, Male, 33 years, KII)

### Shame and stigma

Shame and stigma prevent parents and youths from openly communicating about important SRH topics. Many youths fear that talking about these issues will lead to judgment or condemnation from their family members, causing them to stay silent and avoid seeking help or guidance. This can have devastating consequences for youths suffering in silence after experiencing abuse or rape, unable to break the cycle of pain and trauma on their own. The refusal to talk openly about SRH only perpetuates the stigma and shame, further isolating individuals and hindering their access to vital information and support. One of the FGD participants said:

“…They [parents] are unwilling to provide information based on our developmental stage. If I talk to any male in our village, my father starts asking questions like, Who is that person? What is your relationship? What are you talking about?’ As a result, I feel ashamed to communicate with any male, even about simple issues.” (Youth, Female, 16 years, FGD)

Another participant also stated that

“…Many of our parents perceive that children who want to know about SRH issues exhibit unhealthy behaviors that could lead them to risky choices. As a result, they often think in a punitive, frightening, and authoritarian manner, which makes us prefer to remain silent.” (Youth, Male, 18 years, FGD)

### Parental attitudes towards SRH

Most parents feel it is not important to discuss SRH issues with their children. They think that if the discussion is had with their children, their children will consider practicing the behavior. One of the FGD participants said

“…If I discuss the issue of SRH, the next day they want to practice it. I prefer not to talk about it. They know that it's better to remain silent until they mature.” (Parent, Female, 50 years, FGD)

A KII participant, however, expressed the importance of developing communication from childhood:

“SRH discussions should begin in early childhood and continue into adulthood. If addressed earlier, young people will feel more comfortable talking about it…” (School director, Male, 55 years, KII).

## Discussion

This study highlights several barriers to SRH communication between parents and youths, including a lack of knowledge, cultural and religious beliefs, excessive social media use, shame and stigma, and parental attitudes towards SRH. Many parents struggle to discuss SRH with their children due to a lack of knowledge and confidence, leading to avoidance of these important conversations. Cultural and religious beliefs often deem SRH topics taboo, further inhibiting open communication about SRH within the family and community. Excessive exposure to social media can also hinder meaningful discussions about SRH, as children may trust online sources more than their parents. Additionally, shame and stigma surrounding SRH topics can prevent both parents and youths from openly communicating, potentially leading to harmful consequences.

### Parents’ lack of knowledge

This study found that the parents in this study area lacked the knowledge, skills, and confidence to discuss sexual and reproductive health with their children. Similar findings have been reported in studies conducted in different countries around the world. For example, a study in the United States found that parents often struggle to talk to their children about sexual intercourse due to a lack of knowledge and discomfort (19). Another study in Uganda also found that parents lacked knowledge and confidence in discussing sexual and reproductive health with their children (20). These findings suggest that parents face similar challenges globally when it comes to having important SRH conversations with their children.

### Cultural and religious beliefs

Cultural and religious beliefs make it taboo for youths to speak to their parents about sexual and reproductive health matters. Similarly, studies (4, 18–20) have highlighted how cultural values and religious teachings hinder communication about SRH topics between parents and youth, leading to inadequate information sharing. These findings suggest that various cultures and religious beliefs pose common challenges to open discussions about SRH issues within families.

### Effect of social media use

The major barrier to parent-youth sexual and reproductive health communication was excessive social media use among youths. Youths believe the information they receive from social media, such as Facebook, YouTube, and other media formats, is more valuable than that from their parents. Thus, this can potentially affect parent-youth communication about sexual and reproductive health matters. This finding is corroborated by different studies conducted worldwide (21, 22).

### Shame and stigma

The findings of this study emphasize that sexual shame and stigma are barriers to open communication about SRH issues. Many youths feel that discussing SRH issues with their families is shameful and may lead to stigma. Similarly, studies in India, Sri Lanka, Kenya, and Ethiopia highlighted that stigma and shame are barriers to SRH communication among youths, making them more vulnerable to sexual abuse (12, 23–25). Moreover, a study conducted in Australia found that a culture of open communication and support reduced shame and stigma, enabling young people to openly discuss their SRH concerns (26).

### Parental attitudes towards SRH

The study's findings showed that most parents have negative views about discussing SRH issues with their children. Judgmental attitudes, parental apprehension, and a lack of openness to dialogue are significant barriers to communication between parents and youths regarding SRH. Judgmental attitudes, where parents view certain topics as taboo or morally wrong, can create discomfort, leading youths to avoid communication about SRH. Additionally, a lack of openness to dialogue, characterized by a reluctance to listen to and engage with their child's perspectives, can perpetuate feelings of disconnection and isolation, making it difficult for youths to feel comfortable discussing SRH issues with their parents. Consequently, these factors can hinder the development of healthy communication. The findings of our study corroborated with different studies conducted in Tanzania, Ghana, and Ethiopia (21, 26–29).

### Strengths and limitations

This qualitative study has several strengths and limitations. One of its strengths is data triangulation from KIIs, in-depth interviews (IDIs), and FGDs with various participants, including parents, youths, and teachers. However, a limitation of this study is that it included only four schools (two private and two public). Additionally, the study is susceptible to subjectivity, as the results may be influenced by the researcher's biases and perspectives, affecting the objectivity of the findings.

## Conclusions

This study elucidates various barriers hindering communication about SRH between parents and youths. These barriers include parental lack of knowledge, cultural and religious beliefs, the effect of social media use, shame and stigma, and parental attitudes towards SRH. Therefore, there is a critical need for more community-based actions aimed at educating parents about SRH issues. Empowering parents with accurate information can result in increasing knowledge, changing attitudes, and fostering effective SRH communication among youths and their parents. Using existing community-based organizations, such as religious institutions and schools, to reach out to youths and parents can be an effective method to break down cultural and religious barriers and avoid shame and stigma.

Community and school-level discussions can create a more inclusive and supportive environment where youths feel confident and can seek guidance and support for SRH issues. Using mass media, health talks, and print materials to raise awareness of the necessity of SRH communication can help bridge the gap between youths and their parents. High schools should incorporate regular SRH education sessions and invite healthcare providers to increase students’ knowledge and change perceptions. Strengthening peer education clubs in schools can maximize interpersonal communication, and the skills gained can ease communication with parents and teachers.

## Data Availability

The original contributions presented in the study are included in the article/Supplementary Material, further inquiries can be directed to the corresponding author.
